# AT-MSCs Antifibrotic Activity is Improved by Eugenol through Modulation of TGF-β/Smad Signaling Pathway in Rats

**DOI:** 10.3390/molecules25020348

**Published:** 2020-01-15

**Authors:** Moustafa Fathy, Motonori Okabe, Heba M. Saad Eldien, Toshiko Yoshida

**Affiliations:** 1 Department of Regenerative Medicine, Graduate School of Medicine and Pharmaceutical Sciences, University of Toyama, Toyama 930-0194, Japan; moustafa_fathyy@yahoo.com (M.F.); okabe@med.u-toyama.ac.jp (M.O.); 2 Department of Biochemistry, Faculty of Pharmacy, Minia University, Minia 61519, Egypt; 3 Department of Anatomy, College of Medicine, Jouf University, Jouf 74311, Saudi Arabia; hebasaadeldien2015@yahoo.com; 4 Department of Histology and Cell Biology, Faculty of Medicine, Assiut University, Assiut 71515, Egypt

**Keywords:** AT-MSCs, eugenol, hepatic fibrosis, TGF-β, Smad3, TNF-α, IL-6, *α*-SMA

## Abstract

For hepatic failure, stem cell transplantation has been chosen as an alternative therapy, especially for mesenchymal stem cells (MSCs). The aim of this study was to investigate the effect of eugenol (EUG) on the in vivo antifibrotic activity of adipose tissue-derived MSCs (AT-MSCs) and the underlying mechanism. After characterization of MSCs, rats were divided into five groups, Group 1 (normal control), Group 2 (CCl_4_), Group 3 (CCl_4_ + AT-MSCs), Group 4 (CCl_4_ + EUG) and Group 5 (CCl_4_ + AT-MSCs + EUG). Biochemical and histopathological investigations were performed. Furthermore, expression of type 1 collagen, α-SMA, TGF-β1, Smad3 and P-Smad3 was estimated. Compared to the single treatment with AT-MSCs, the combination treatment of the fibrotic rats with AT-MSCs and EUG significantly improved the plasma fibrinogen concentration, IL-10 level and proliferating cell nuclear antigen expression, and also significantly decreased the serum levels of liver enzymes, IL-6, IL-1β, TNF-α, type III collagen, hyaluronic acid, hydroxyproline and the TGF-β growth factor. Furthermore, the combination treatment significantly decreased the hepatic expression of fibrotic markers genes (*Type 1 collagen* and *α-SMA*) and proteins (α-SMA, TGF-β1 and phospho-Smad3) more than the treatment with AT-MSCs alone. We demonstrated that the combination treatment with EUG and AT-MSCs strongly inhibited the advancement of CCl_4_-induced hepatic fibrosis, compared with AT-MSCs alone, through TGF-β/Smad pathway inhibition. This approach is completely novel, so more investigations are necessary to improve our perception of the underlying molecular mechanisms accountable for the effects of EUG on the antifibrotic potential of AT-MSCs.

## 1. Introduction

Recently, for hepatic failure, stem cell transplantation has been chosen as an alternative therapy. Mesenchymal stem cells (MSCs), derived from different organs, have much superiority in regenerative medicine, including multipotent differentiation capacity, their low immunogenic properties and high proliferation rate. Moreover, they have the ability to drift toward the microenvironments of injured sites [1,2,3]. MSCs also present many other features, like no adverse reactions related to MSCs transplant, and long-term storage without considerable loss of potency [4]. They are immunomodulatory, can directly differentiate to new hepatocytes, have antifibrotic properties, can promote tissue repair and also inhibit hepatic stellate cells activation [5].

It was previously reported that bone marrow-derived mesenchymal stem cells (BM-MSCs) are capable of attenuating liver fibrosis [6]. Adipose tissue (AT) is also a good provenance of high proliferative potential MSCs. Clinical investigations using AT-derived MSCs (AT-MSCs) showed enhanced liver function and histology [7,8,9]. Furthermore, the easy arrival to subcutaneous AT, its uncomplicated isolation methods and the reproducibility to sample this tissue, make AT the most appealing source of MSCs [10].

Hepatic fibrosis is a common outcome of chronic hepatic disorders. During hepatic fibrogenesis, there are raised inflammatory responses and extravagant accumulation of the hepatic extracellular matrix. If inappropriately handled, hepatic fibrosis may proceed to cirrhosis, resulting in patients requiring hepatic transplantation [11,12]. At the present time, hepatic transplantation is the only available therapeutic choice for those patients, but its application is limited by high cost, organ shortage, and the necessity of lifelong immunosuppressive therapy to avoid transplant rejection [13]. Therefore, novel therapeutic strategies are needed to improve care for such patients and for the treatment of hepatic fibrosis.

Recently, we demonstrated the antifibrotic effect of eugenol (EUG), which is the active ingredient of clove essential oil, through its in vivo attenuation of the inducible nitric oxide synthase pathway [14]. Furthermore, we previously maintained and enhanced the differentiation potential of various tissues-derived stem cells [2,3,15,16,17]. As an extension of the results of previous studies, the present study was designed to inspect the effect of eugenol on the in vivo antifibrotic activity of AT-MSCs and to investigate the underlying mechanisms. Thus, our aim was to examine the effect of eugenol on the intravenously-administered AT-MSCs in improving liver function and decreasing hepatic fibrosis in a carbon tetrachloride (CCl_4_)-induced hepatic fibrosis rat model.

## 2. Results 

### 2.1. Cell Surface Markers Expression

**Figure 1:**
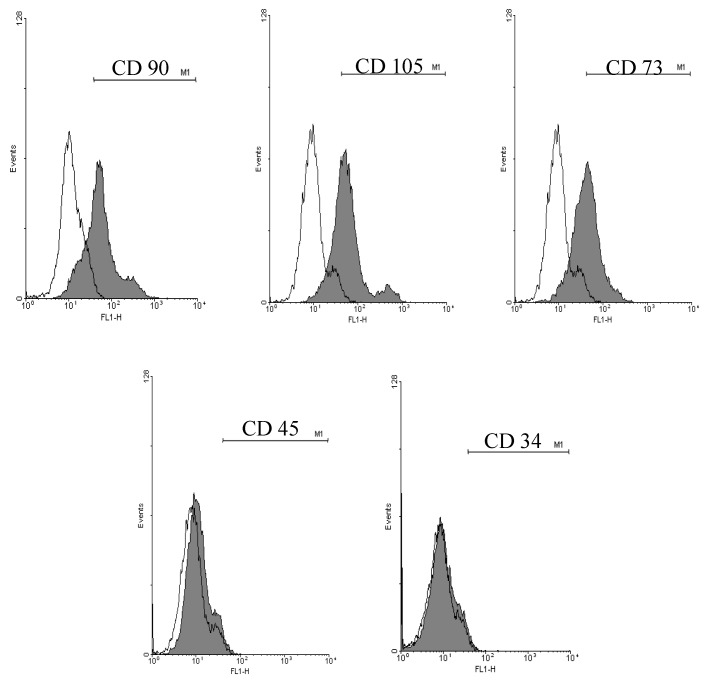
Cell surface markers expression of adipose tissue-derived mesenchymal stem cells (AT-MSCs). Representative histograms of the flow cytometric analysis of the isolated AT-MSCs for the expression of mesenchymal stem cells (MSCs) markers (CD90, CD105 and CD73) and hematopoietic markers (CD34 and CD45). AT-MSCs: Adipose tissue-derived mesenchymal stem cells.

Cell surface markers expression of AT-MSCs was examined by flow cytometric (FCM) analysis for MSC markers (CD90, CD105 and CD73) and hematopoietic markers (CD34 and CD45). As shown in Figure 1, 75.8% ± 3.5%, 82.8% ± 4.9% and 80.5% ± 5.1% of the cultured AT-MSCs were expressed CD90, CD105 and CD73, respectively. On the other hand, only 0.5% ± 0.03% and 4.3% ± 0.3% of them expressed the hematopoietic markers CD34 and CD45, respectively.

### 2.2. Prothrombin Time and Fibrinogen Concentration Assay

**Figure 2:**
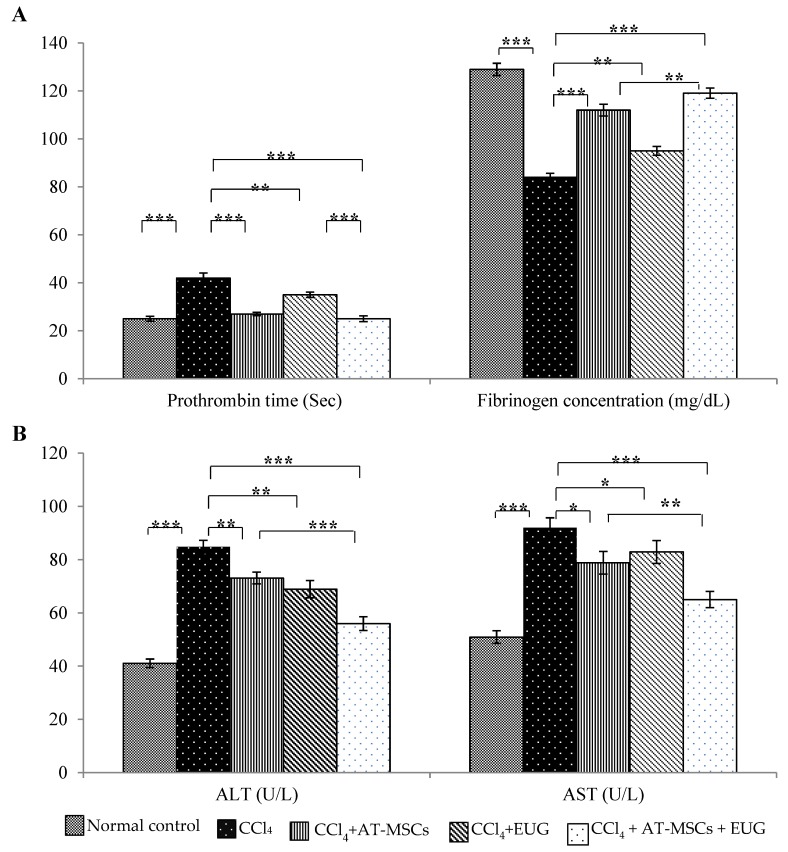
Prothrombin time, fibrinogen concentration and liver enzymes levels. (**A**) Prothrombin time (sec) and plasma fibrinogen concentration (mg/dL) for the different groups. (**B**) Serum liver enzymes (ALT and AST) levels (U/L) for the different groups. Bars represent mean ± standard deviation (SD). Significant difference between groups is analyzed by one-way analysis of variance (ANOVA) test, where: *; *p* < 0.05, **; *p* < 0.01, ***; *p* < 0.001. AT-MSCs: Adipose tissue-derived mesenchymal stem cells, EUG: Eugenol.

The prothrombin time of normal control rats was 25 ± 1.0 sec. CCl_4_ significantly (*p* < 0.001) increased the prothrombin time to 42 ± 2.1 sec while treatment with AT-MSCs, EUG or combination of them significantly decreased it to 27 ± 0.7, 35 ± 1.1 or 25 ± 1.2 sec, respectively, when compared to CCl_4_ group, as shown in Figure 2A.

Also, Figure 2A showed that CCl_4_ significantly (*p* < 0.001) decreased plasma fibrinogen concentration to 83.9 ± 1.7 mg/dL when compared to normal control group 128.9 ± 2.5 mg/dL. Treatment with AT-MSCs, EUG or both of them significantly increased the fibrinogen concentration to 111.9 ± 2.4, 94.9 ± 1.8 or 119.1 ± 2.1 mg/dL, respectively. There is a significant difference (*p* < 0.01) between rats treated with AD-MSCs + EUG and rats treated with AD-MSCs alone.

### 2.3. Liver Enzymes (ALT and AST) Levels

Serum levels of ALT and AST were significantly (*p* < 0.001) increased by CCl_4_ when compared to normal control rats. Both enzymes levels were significantly decreased when rats treated with AT-MSCs, EUG or both of them when compared to CCl_4_ group. In addition, ALT and AST levels were significantly (*p* < 0.001 for ALT and *p* < 0.01 for AST) decreased in CCl_4_ + AT-MSCs + EUG group compared to CCl_4_ + AT-MSCs group (Figure 2B).

### 2.4. Serum Inflammatory Cytokines (IL-1β, TNF-α, IL-6 and IL-10) Levels

**Figure 3:**
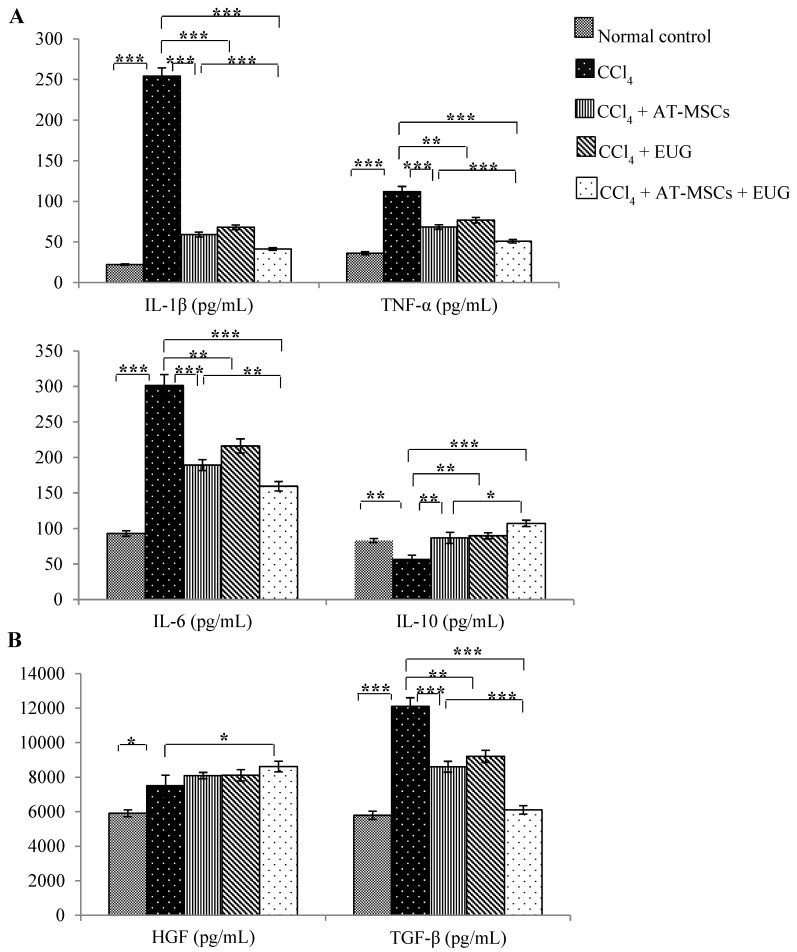
Serum inflammatory cytokines and growth factors levels. (**A**) Serum cytokines (IL-1β, TNF-α, IL-6 and IL-10) levels (pg/mL) for the different groups. (**B**) Serum growth factors (HGF and TGF-β) levels (pg/mL) for the different groups. Bars represent mean ± SD. Significant difference between groups is analyzed by one-way ANOVA test, where: *; *p* < 0.05, **; *p* < 0.01, ***; *p* < 0.001. AT-MSCs: Adipose tissue-derived mesenchymal stem cells, EUG: Eugenol, IL-1β: Interlukin-1β; IL-6: Interlukin-6; IL-10: Interlukin-10; TNF-α: Tumor necrosis factor-α; HGF: Hepatocyte growth factor; TGF-β: transforming growth factor-β.

As shown in Figure 3A, the measured pro-inflammatory cytokines (interleukin-1beta (IL-1β), tumor necrosis factor-alpha (TNF-α) and interleukin-6 (IL-6)) were significantly (*p* < 0.001) increased with CCl_4_ when compared to normal control group. Treatment with AT-MSCs, EUG or both of them significantly decreased these cytokines levels compared to the CCl_4_ group. Furthermore, their serum levels were significantly decreased in rats of the CCl_4_ + AT-MSCs + EUG group compared to rats of the CCl_4_ + AT-MSCs group. On the other hand, the interleukin-10 (IL-10) serum level was significantly (*p* < 0.01) decreased with CCl_4_, while the treatment with AT-MSCs, EUG or both of them significantly increased its level compared to the CCl_4_ group, and there is a significant (*p* < 0.05) difference between CCl_4_ + AT-MSCs and CCl_4_ + AT-MSCs + EUG groups.

### 2.5. Serum Growth Factors (Hepatocyte Growth Factor (HGF) and Transforming Growth Factor-Beta (TGF-β)) Levels

Both factors were significantly (*p* < 0.05 for HGF and *p* < 0.001 for TGF-β) increased with CCl_4_ when compared to the normal control group (Figure 3B). Serum levels of HGF were significantly (*p* < 0.05) increased only in rats treated with AT-MSCs + EUG compared to the CCl_4_ group, and there is no significant difference between CCl_4_ + AT-MSCs and CCl_4_ + AT-MSCs + EUG groups.

TGF-β serum levels were significantly decreased in rats treated with AT-MSCs, EUG or both of them compared to CCl_4_ rats. In addition, there is a significant (*p* < 0.001) difference between CCl_4_ + AT-MSCs and CCl_4_ + AT-MSCs + EUG groups.

### 2.6. Levels of Type III Collagen, Hyaluronic Acid and Hepatic Hydroxyproline Content

**Figure 4:**
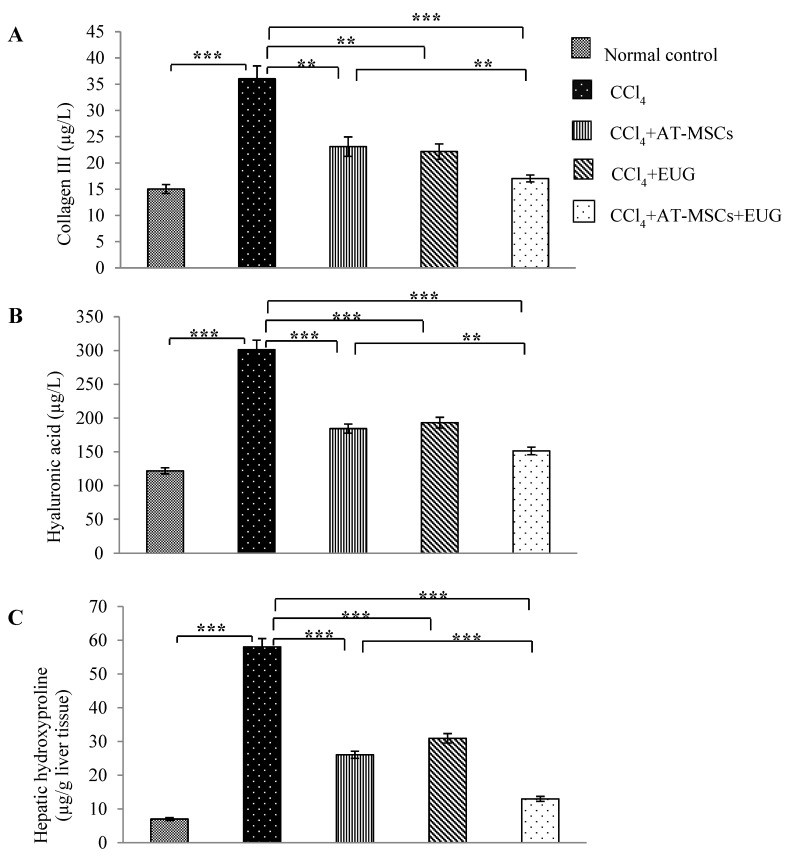
Levels of type III collagen, hyaluronic acid and hepatic hydroxyproline content. Serum levels of (**A**) collagen III and (**B**) hyaluronic acid (µg/L) for the different groups. (**C**) Hepatic hydroxyproline content (µg/g liver protein) for the different groups. Bars represent mean ± SD. Significant difference between groups is analyzed by one-way ANOVA test, where: **; *p* < 0.01, ***; *p* < 0.001. AT-MSCs: Adipose tissue-derived mesenchymal stem cells, EUG: Eugenol.

Figure 4 showed that, in addition to hepatic hydroxyproline content, serum levels of type III collagen and hyaluronic acid were significantly (*p* < 0.001) increased with CCl_4_ when compared to the normal control group. Also, they were significantly decreased in rats treated with AT-MSCs, EUG or both of them compared to CCl_4_ rats. Furthermore, there is a significant difference between CCl_4_ + AT-MSCs and CCl_4_ + AT-MSCs + EUG groups (*p* < 0.001 for hepatic hydroxyproline content and *p* < 0.01 for type III collagen and hyaluronic acid serum levels).

### 2.7. Expression of Proliferating Cell Nuclear Antigen (PCNA)

**Figure 5:**
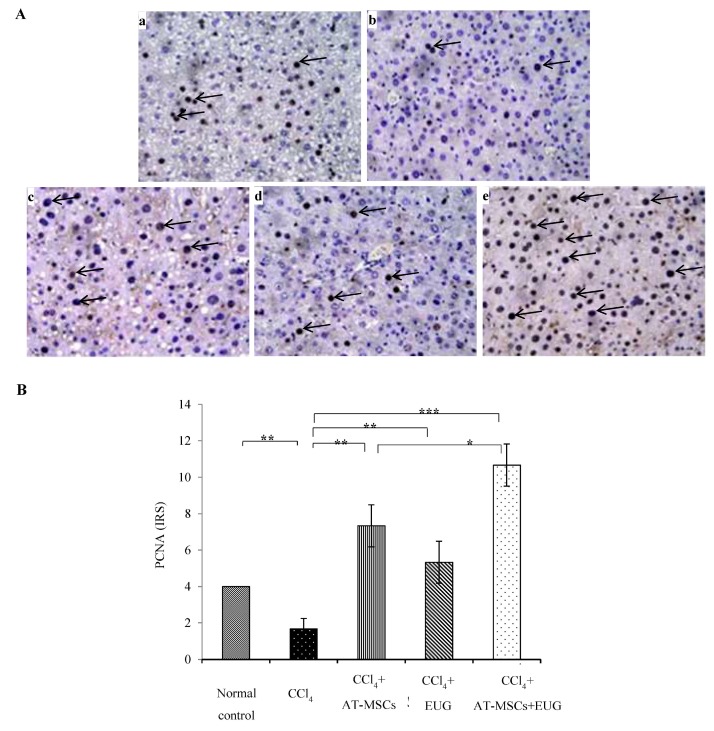
Expression of the proliferating cell nuclear antigen. (**A**) Representative photos of PCNA staining of hepatic tissues of: (a) normal control rats, (b) fibrotic rats which received CCl_4_, (c) rats that received CCl_4_ + AT-MSCs, (d) rats receiving CCl_4_ + EUG, (e) rats who received CCl_4_ + AT-MSCs + EUG, (Magnification: x400). (**B**) Staining intensities were expressed in immuno-reactive score (IRS). Bars represent mean ± SD. Significant difference between groups is analyzed by one-way ANOVA test, where: *; *p* < 0.05, **; *p* < 0.01, ***; *p* < 0.001. AT-MSCs: Adipose tissue-derived mesenchymal stem cells, EUG: Eugenol, PCNA: Proliferating cell nuclear antigen, IRS: Immuno-reactive score.

Rats receiving CCl_4_ showed significant (*p* < 0.01) low expression of PCNA compared to normal control rats. Its expression was significantly (*p* < 0.01 for rats treated with AT-MSCs or EUG alone, and *p* < 0.001 for rats treated with AT-MSCs + EUG) increased when compared to the CCl_4_ group. There is a significant (*p* < 0.05) difference between CCl_4_ + AT-MSCs and CCl_4_ + AT-MSCs + EUG groups (Figure 5).

### 2.8. Type 1 Collagen, α-Smooth Muscle Actin (α-SMA) and TGF-β1 Genes Expression

**Figure 6:**
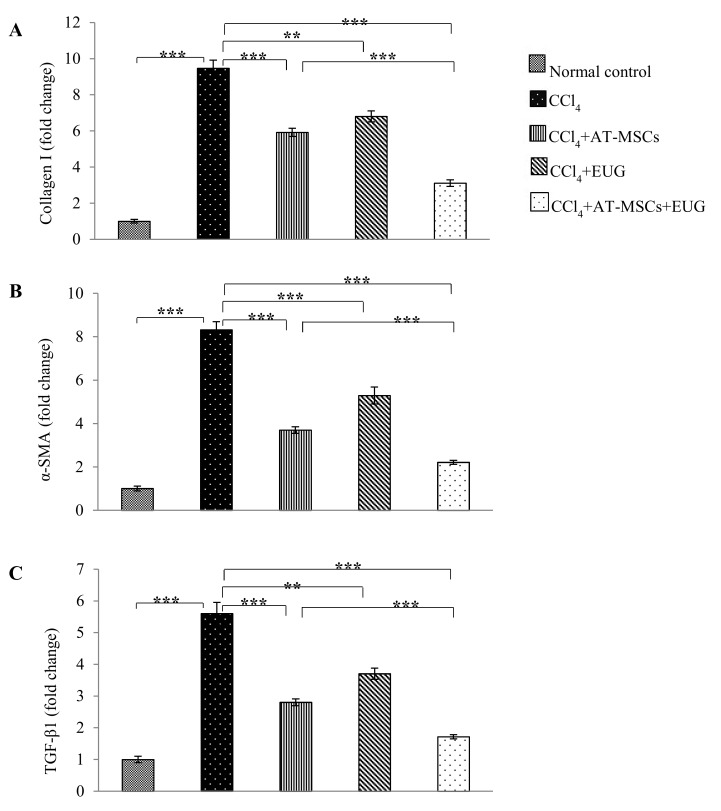
Expression of *Type 1 collagen*, *α-SMA* and *TGF-β1* genes. Quantitative real time polymerase chain reaction (RT-PCR) was used to estimate the expression of (**A**) *Type 1 collagen*, (**B**) *α-SMA* and (**C**) *TGF-β1* genes in hepatic tissues for the different groups. Fold change relative to the expression of normal control group and normalized to the internal control glyceraldehyde 3-phosphate dehydrogenase (*GAPDH*) was shown. Bars represent mean ± SD. Significant difference between groups is analyzed by one-way ANOVA test, where: **; *p* < 0.01 and ***; *p* < 0.001. AT-MSCs: Adipose tissue-derived mesenchymal stem cells, EUG: Eugenol, *α-SMA*: α-smooth muscle actin, *TGF-β1*: Transforming growth factor-β, *GAPDH*: glyceraldehyde 3-phosphate dehydrogenase.

Relative to rats of the normal control group and after normalization to glyceraldehyde 3-phosphate dehydrogenase (*GAPDH*) as a housekeeping gene, Figure 6 showed that CCl_4_ significantly (*p* < 0.001) increased the hepatic mRNA levels of *Type 1 collagen*, *α-SMA* and *TGF-β1* genes to become 9.4 ± 0.4 folds, 8.3 ± 0.3 folds and 5.6 ± 0.3 folds, respectively, those of the normal control group. Also, the hepatic expression of the three genes was significantly decreased when rats treated with AT-MSCs, EUG or both of them when compared to the CCl_4_ group. More interestingly, there is a significant (*p* < 0.001) difference between CCl_4_ + AT-MSCs and CCl_4_ + AT-MSCs + EUG groups in the expression of the examined genes.

### 2.9. Expression of α-SMA, TGF-β1 and Phospho-Smad3 Proteins

**Figure 7:**
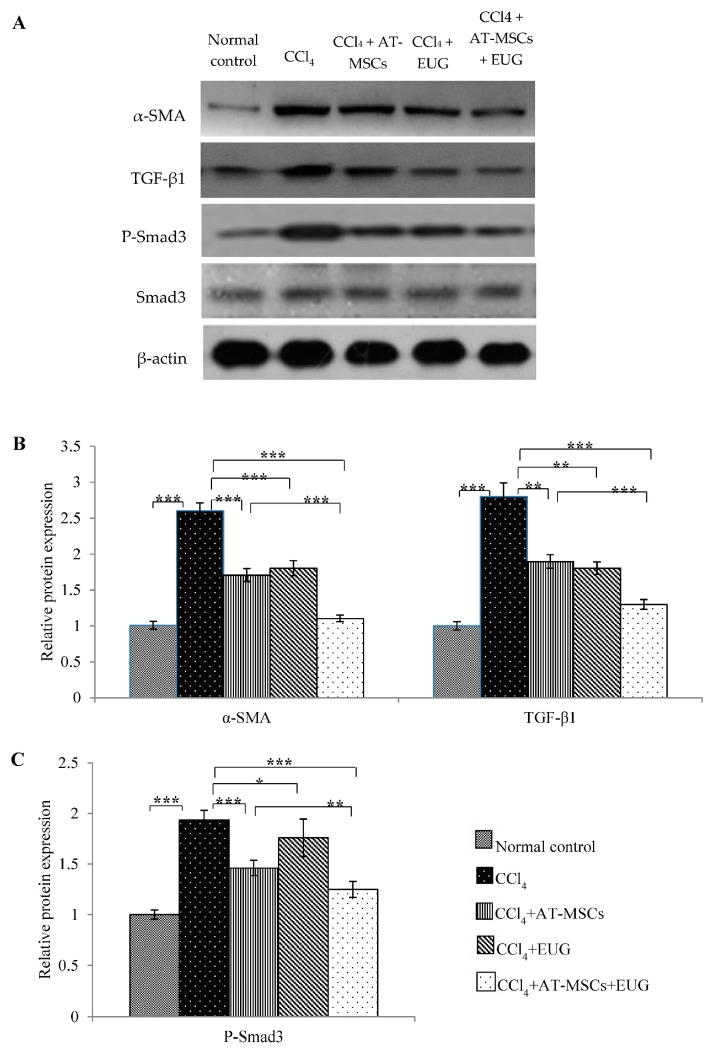
Expression of α-SMA, TGF-β1 and phospho-Smad3 proteins. (**A**) Representative immunoprecipitation blots of α-SMA, TGF-β1, Smad3 and P-Smad3 proteins for the different groups. (**B**) Relative expression of α-SMA and TGF-β1 proteins for the different groups, normalized to β-actin expression. (**C**) Relative expression of P-Smad3 protein for the different groups, normalized to β-actin and Smad3 proteins expression. Bars represent mean ± SD. Significant difference between groups is analyzed by one-way ANOVA test, where: *; *p* < 0.05, **; *p* < 0.01, ***; *p* < 0.001. AT-MSCs: Adipose tissue-derived mesenchymal stem cells, EUG: Eugenol, α-SMA: α-smooth muscle actin, TGF-β1: Transforming growth factor-β.

Hepatic expression of α-SMA, TGF-β1, Smad3 and phospho-Smad3 (P-Smad3) proteins was estimated by Western blot (Figure 7A). After normalization to β-actin protein expression and relative to that of the normal control group, the relative α-SMA and TGF-β1 proteins expression was 2.6 ± 0.1 and 2.8 ± 0.1 folds, respectively, in the CCl_4_ group, as shown in Figure 7B. Hepatic expression of both proteins was significantly decreased when rats were treated with AT-MSCs, EUG or both of them when compared to the CCl_4_ group. In addition, there is a significant (*p* < 0.001) difference between CCl_4_ + AT-MSCs and CCl_4_ + AT-MSCs + EUG groups.

Figure 7C showed, after normalization to β-actin and Smad3 proteins expression, that P-Smad3 protein expression was significantly (*p* < 0.001) increased by CCl_4_ compared to the normal control group. Furthermore, the treatment with AT-MSCs, EUG or both of them significantly decrease its expression compared to the CCl_4_ group. Furthermore, there is a significant (*p* < 0.01) difference between CCl_4_ + AT-MSCs and CCl_4_ + AT-MSCs + EUG groups.

### 2.10. Hepatic Histopathological Changes

**Figure 8:**
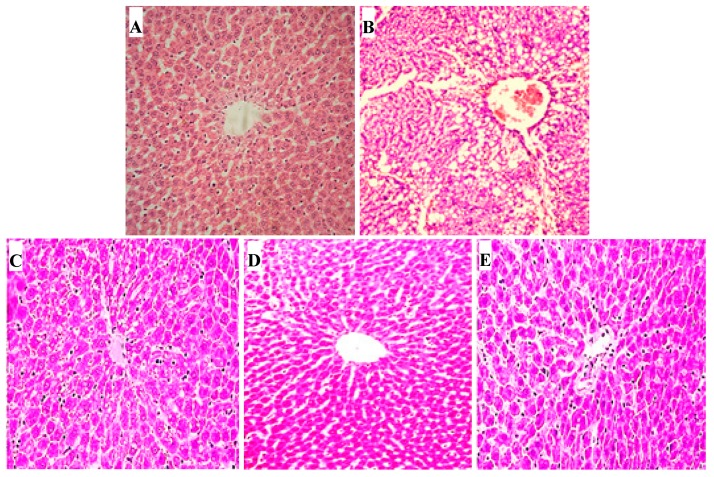
Histopathological examination for hepatic tissues. Hematoxylin-eosin (H&E)-stained hepatic sections of: (**A**) normal control rats, (**B**) fibrotic rats which received CCl_4_, (**C**) rats receiving CCl_4_ + AT-MSCs, (**D**) rats who received CCl_4_ + EUG, (**E**) rats that received CCl_4_ + AT-MSCs + EUG, (Magnification: ×100). AT-MSCs: Adipose tissue-derived mesenchymal stem cells, EUG: Eugenol.

For normal control rats, hepatic histopathological investigation revealed no fatty changes or inflammatory infiltration and normal hepatic architecture. Hepatic sections of the CCl_4_ fibrotic group showed disarrangement of hepatocytes, fatty degeneration, necrosis and vacuole formation. Compared to CCl_4_ fibrotic rats, hepatic sections of rats treated with AT-MSCs or EUG showed enhanced changes, as the absence of vacuoles and necrosis, but moderate spaces between sinusoids were shown. In hepatic sections of rats treated with AT-MSCs and EUG, there is no obvious histological injury with reduced sinusoidal spaces and normal hepatocytes with distinct nuclei (Figure 8).

## 3. Discussion

Recently, for treating hepatic disease, stem cell-based treatment has been suggested as an appropriate alternative strategy. During the past few years, researchers have reported that MSCs counter and exert therapeutic benefits against hepatic fibrosis [18,19]. Furthermore, the development of new approaches enhancing the MSCs therapy may be helpful to end-stage liver fibrosis patients.

It was reported that EUG exerted various pharmacological activities [20], and showed a hepatoprotective effect by improving the hepatic antioxidant capacity and declining the inflammation via the downregulation of the iNOS pathway [14]. Therefore, we hypothesized that treatment by AT-MSCs with EUG would yield an utmost antifibrotic effect and improvement in hepatic function compared with treatment with AT-MSCs alone.

MSCs obtained from AT were characterized for the expression of surface markers by FCM. They highly expressed the MSCs markers (CD90, CD105 and CD73) and showed low expression of the hematopoietic markers (CD45 and CD34). In the present study, compared to the treatment with AT-MSCs alone, the combination treatment of the fibrotic rats with AT-MSCs and EUG significantly improved the plasma fibrinogen concentration, anti-inflammatory cytokine (IL-10) level and PCNA expression, and also significantly decreased the serum levels of liver enzymes (ALT and AST), inflammatory cytokines (TNF-α, IL-1β and IL-6), fibrotic markers (type III collagen, hyaluronic acid, hydroxyproline) and the TGF-β growth factor. Furthermore, the combination treatment also significantly decreased the hepatic expression of fibrotic markers genes (*Type 1 collagen* and *α-SMA*) and proteins (α-SMA, TGF-β1 and phospho-Smad3) more than the treatment with AT-MSCs alone.

Wound healing consists of many phases, including hemostasis, which involves blood coagulation. Blood coagulation ability could be measured by fibrinogen concentration and prothrombin time, which measures the blood coagulation extrinsic pathway [21]. In a hepatic fibrotic animal model, we have assessed the potential of the combination treatment with AT-MSCs and EUG on the hemostatic mechanism, for the first time, by attention to prothrombin time and fibrinogen concentration. This combination may enhance the hemostatic mechanism by stimulating the secretion of many growth factors and cytokines which help regulate hepatic cellular responses, stimulate the differentiation of endogenous tissue progenitors and the repair of hepatic tissues, and play an important role in hemostasis.

Fibrosis, in this study, was confirmed by measuring the liver enzymes level and a histopathology study. Hepatic tissues of fibrotic rats treated with AT-MSCs and EUG showed great improvements, compared with the fibrotic group, with the evanescence of necrosis and fatty changes which was near to the normal control hepatic sections. The combination treatment showed more antifibrotic effects than treatment with AT-MSCs alone, indicating the synergistic effects between AT-MSCs and EUG.

Hepatic damage, inflammation and fibrosis is always accompanied with elevated levels of hepatic enzymes and pro-inflammatory cytokines. The combination treatment of the fibrotic rats with AT-MSCs and EUG significantly reduced inflammatory reactions, diminished the levels of liver enzymes and the pro-inflammatory cytokines (TNF-*α*, Il-1*β*, and Il-6), and increased the anti-inflammatory cytokine IL-10 level, reflecting the potent anti-inflammatory effect. Also, downregulation of the fibrotic genes (*Type 1 collagen* and *α-SMA*) and the growth factor (TGF-*β1*) expression, which was evaluated by quantitative RT-PCR, was significantly associated with the combination treatment more than that with the single treatment with AT-MSCs, indicating decreased hepatic fibrosis. Furthermore, the serum level of HGF was significantly increased, compared to the fibrotic group, only with the combination treatment, indicating repair of hepatic tissues and tissue remodeling.

Extracellular matrix (ECM) remodeling and collagen turnover are regulated by many matrix metalloproteinases (MMPs) and tissue inhibitors of metalloproteinases (TIMPs). During the process of fibrosis resolution, there is an increase in hepatic stellate cells apoptosis and collagenase activity; also there is a diminution in TIMP expression. It was reported that MSCs decreased TIMP-1 expression or increased the expression of MMPs in fibrotic models [22,23,24]. In this study, as well documented markers of fibrosis, levels of type III collagen, hyaluronic acid and hepatic hydroxyproline content, were estimated. Our results showed that levels of these fibrotic markers were significantly diminished, especially when the combination treatment was used. EUG might improve the antifibrotic effect of AT-MSCs by different mechanisms; it might increase the ability of AT-MSCs to suppress the pathological process initiated by inflammation, or increase AT-MSCs fusion with hepatocytes, which may contribute to microenvironment modification and differentiation into hepatocytes, leading to increased tissue regeneration and decreased fibrosis. Furthermore, EUG might increase the homing capacity of AT-MSCs for the injured liver, leading to more hepatocyte differentiation, resulting in increased hepatic PCNA expression in fibrotic rats treated with AT-MSCs and EUG, more than those treated with AT-MSCs alone. 

More interestingly, EUG, through its anti-inflammatory effect, might improve the immune-modulating activity of MSCs, which have the ability to increase the anti-inflammatory phenotype of macrophages, leading to increase the pro-regenerative factors levels.

Moreover, this study showed the hepatic up-regulation of α-SMA and TGF-β1 proteins and the phosphorylation of Smad3 protein in fibrotic rats. This upregulation was diminished by the combination treatment of AT-MSCs and EUG more than the single treatment with EUG. Actually, for hepatic fibrosis, the TGF-β signaling pathway could be considered to be a promising therapeutic target. TGF-β1, which is an important mediator for the progression of hepatic fibrosis, implements its fibrogenic functions, such as collagen synthesis, via the Smad pathway by the transmembrane TGF-β receptors activation [25,26,27]. It was reported that the inhibition of TGF-β1 suppressed the formation of 4-hydroxynonenal, which is a stable end product of lipid peroxidation and a factor of fibrotic processes, leading to mitotic activity restoration and the reduction of apoptosis in rat hepatocytes [28]. In this study, we found that, in fibrotic rats, AT-MSCs + EUG more strongly inhibited the TGF-β/Smad signaling pathway through the inhibition of Smad phosphorylation and the expression of TGF-β1 and α-SMA.

These data clearly showed the synergistic effect of EUG with AT-MSCs, which may represent a novel therapeutic approach for treating hepatic fibrosis, and in regenerative medicine. This approach is completely novel, to the best of our knowledge, so more investigations are necessary and currently going on, in order to enhance our understanding of the underlying molecular mechanisms accountable for the EUG effects on the antifibrotic potential of AT-MSCs, and to increase knowledge concerning the capability of our concept to be clinically viable.

## 4. Materials and Methods

### 4.1. Drugs and Reagents

All chemicals were purchased from commercial suppliers and were of analytical grade. Eugenol (99.0%, Sigma Aldrich Company, St Louis, Missouri, USA) was prepared 1:1 (*v*/*v*) in olive oil at a concentration 10 mg/mL, stored in the dark at 4 °C and equilibrated at room temperature before use. Carbon tetrachloride (CCl_4_, Wako Pure Chemical Industries, Osaka, Japan) was diluted in olive oil 1:1 (*v*/*v*).

### 4.2. Isolation, Culture and Characterization of AT-MSCs

MSCs were obtained from the AT of two-month-old male rats (weighting 200 ± 25 g) using the method previously described by *De Luna-Saldivar* et al. [10]. The isolated AT-MSCs were cultured in Dulbecco’s Modified Eagle’s Medium (DMEM, Sigma-Aldrich, St. Louis, MO, USA) containing 10% fetal bovine serum (FBS, Biosolutions International, Melbourne, Australia) and 1% Penicillin-Streptomycin mixture (Nacalai Tesque, Kyoto, Japan) at 37 °C in a 5% CO_2_ humidified incubator. The medium was changed every 3 days. Cells at 80%–85% confluence were trypsinized and passaged until the fourth passage. Then the cells were washed and suspended in PBS for use.

Characteristics of AT-MSCs were confirmed by FCM analysis to ensure that these cells express the MSC markers (CD73, CD105 and CD90), but not the hematopoietic markers (CD34 and CD45). Briefly, after blocking with 5% bovine serum albumin (BSA; Sigma-Aldrich) in PBS for 30 min at room temperature, cells were stained at room temperature for 1 h with fluorescein isothiocyanate (FITC)- or phycoerythrin (PE)-conjugated antibodies at a concentration of 20 µL/1 × 10^6^ cells. Antibodies against CD90, CD105, CD34, CD45 (Beckman Coulter, Brea, CA, USA) and CD73 (BD Pharmingen, Franklin Lakes, NJ, USA) were used. Controls were incubated with FITC- or PE-conjugated same isotype control antibodies. The FACSCalibur flow cytometer (Becton Dickinson, Franklin Lakes, NJ, USA) was used for performing flow cytometry using Cell Quest software, and WinMDI ver 2.9 was used for data analysis.

### 4.3. Animals and the Experimental Design

Fifty healthy male Crl: SD-1 (ICR) rats (140 ± 10 g), purchased from Japan SLC (Hamamatsu, Japan), were used. Animal care and study protocols were preceded according to the guidelines established by The Experimental Animal Center and Research Ethics Committee, University of Toyama, Toyama, Japan. Hepatic fibrosis was induced by intraperitoneal (i.p.) injection of rats twice a week with 1 mL/kg CCl_4_ diluted in olive oil 1:1 (*v*/*v*) for six weeks [29]. AT-MSCs (9.75 million cells/kg), at week number three, intravenously (i.v.) injected, in 0.5 mL Plasma-Lyte A, into the tail vein of the rat slowly and cautiously to avoid an embolism [30]. Eugenol diluted in olive oil 1:1 (*v*/*v*) was intraperitonealy (i.p.) injected daily at a dose 10 mg/kg [14].

Rats were randomly divided into five groups:

Group 1 (normal control group): Rats injected only with olive oil.

Group 2 (CCl_4_ group): Rats received CCl_4_ as described above.

Group 3 (CCl_4_ + AT-MSCs group): Rats received CCl_4_ and a single dose of AT-MSCs at week 3 as described above.

Group 4 (CCl_4_ + EUG group): Rats received CCl_4_ and EUG as described above.

Group 5 (CCl_4_ + AT-MSCs + EUG group): Rats received CCl_4_, AT-MSCs and EUG as described above.

At the end of the experiment at week 7, after fasting for 12 h, the rats were anesthetized and blood samples were collected. Then, rats were sacrificed and liver samples were excised, washed and collected for analysis.

### 4.4. Prothrombin Time and Fibrinogen Concentration Assay

Using Coagpia™ PT-N kit (SEKISUI MEDICAL CO., LTD., Tokyo, Japan) according to manufacturer’s instructions, prothrombin time was estimated. After blood collection in sodium citrate-containing centrifuge tubes, plasma was obtained after centrifugation. Briefly, to 100 µL of plasma placed in a 37 °C water bath, 200 µL of thromboplastin reagent was added after pre-warming. Then, the time taken for clotting was calculated. Plasma fibrinogen concentration was estimated as described before [30].

### 4.5. Assay of Serum Liver Enzymes, Inflammatory Cytokines and Growth Factors Levels

Serum alanine transaminase (ALT) and aspartate transaminase (AST) enzyme activities were evaluated using commercially available kits purchased from Abcam KK, Tokyo, Japan according to the manufacturer’s instructions. IL-6, IL-10, IL-1β and TNF-α cytokines were estimated by a Luminex analyzer (Merck, Darmstadt, Germany). The levels of growth factors, HGF and TGF-β, were estimated with enzyme-linked immunosorbent assay (ELISA) kits (Abcam KK) using HTS Multi-Mode Microplate Reader (BioTek Instruments, Winooski, VT, USA) according to the manufacturer’s instruction.

### 4.6. Measurement of Serum Levels of Type III Collagen and Hyaluronic Acid and Hepatic Hydroxyproline Content

ELISA kits were used for the determination of serum levels of type III collagen (LifeSpan BioSciences, Inc., Seattle, WA, USA) and hyaluronic acid (AMS Biotechnology Ltd., Abingdon, UK) according to the manufacturer’s instructions.

For measurement of hepatic hydroxyproline content, hepatic tissues were hydrolyzed at 120 °C for 16 h with 6 N HCl. The lysates were cooled and centrifuged for 10 min at 13,000× g after neutralization with 6 N NaOH. Acetate/citrate buffer and 7% chloramine T were added to the supernatants. Then, the mixture was incubated at 60 °C for 35 min with Ehrlich’s solution. The absorbance was measured, after cooling, at 560 nm using HTS Multi-Mode Microplate Reader (BioTek Instruments). For each sample, the hydroxyproline concentration was calculated from the standard curve, prepared by hydroxyproline (Sigma Aldrich, St. Louis, MO, USA). Concentration was expressed as micrograms per gram of hepatic tissue.

### 4.7. Proliferating Cell Nuclear Antigen Expression Determination

For immunohistochemistry, hepatic paraffin-sections were stained with anti-PCNA antibody (1:4000) (Abcam), according to the manufacturer’s instructions, using the Horseradish peroxidase/DAB (ABC) detection immunohistochemistry kit (Abcam, Cambridge, UK). As shown in Table 1, immuno-reactive score (IRS) was obtained by the product of the intensity of staining score (0–3) and the percentage of positive cells score (0–4) [31].

### 4.8. Quantitative Real-Time Polymerase Chain Reaction

Total RNA was isolated from hepatic tissue using Isogen (Nippon Gene, Tokyo, Japan) according to the manufacturer’s protocol. After treatment with deoxyribonuclease I (DNase I, Sigma-Aldrich, Inc,), cDNA was formed using 1 µg total RNA by the PrimeScript™ RT reagent kit (Takara Bio, Kusatsu, Japan) with oligo-dT (Applied Biosystems, Foster City, CA, USA). Transcript levels were measured by real-time PCR using the sequence-specific primers, shown in Table 2. Amplification was performed in an ABI PRISM 7900HT Sequence Detector (Applied Biosystems, Foster City, CA, USA) using SYBR Green PCR Master Mix (Applied Biosystems) according to the manufacturer’s instructions. Glyceraldehyde 3-phosphate dehydrogenase (GAPDH) was used as internal control gene. Gene expression levels were determined using the comparative threshold cycle (ΔΔCt) method after normalization to GAPDH as a housekeeping gene.

### 4.9. Western Blotting Analysis for Hepatic α-SMA, TGF-β1, Smad3 and Phospho-Smad3 Proteins Expression

Parts of the hepatic tissues were homogenized in T-PER protein extraction reagent (Thermo Fisher Scientific Life Sciences, Waltham, MA, USA) using a Tissue Lyser II apparatus (Qiagen GmbH, Haan, Germany). The homogenates were centrifuged and, using a protein assay kit (Bio-Rad Laboratories Inc., Hercules, CA, USA), the protein concentrations of the supernatants were estimated. A total protein of 50 µg of each homogenate was transferred to a polyvinylidene difluoride (PVDF) membrane (EMD Millipore, Billerica, MA, USA), after electrophoresis on a 12% sodium dodecyl sulfate-polyacrylamide (SDS-PAGE) gel. After blocking in a Tris-buffered saline (TBS-T) blocking solution containing 0.05% Tween-20 and 5% (*w*/*v*) non-fat milk for 1 h, membranes were incubated overnight with primary antibodies for α-SMA (42 kDa), TGF-β1 (28 kDa), Smad3 (52 kDa), phospho-Smad3 (55 kDa) (Abcam, 1:1000) and β-actin (42 kDa, Santa Cruz Biotechnology, Santa Cruz, CA, USA) at 4 °C. After washing, membranes were incubated for 1 h at room temperature with horseradish peroxidase-conjugated polyclonal goat anti-rabbit immunoglobulin secondary antibody (Cell Signaling Technology, Danvers, MA, USA). Band visualization was done using an enhanced chemiluminescence detection kit (GE Healthcare, Chicago, IL, USA) according to the manufacturer’s instructions and detected by the image analyzer for luminescent (LAS-4000, Fujifilm Co., Tokyo, Japan). The band intensity was assessed densitometrically, relative to that of the normal control group after normalization to β-actin.

### 4.10. Histopathological Examination

After formalin fixation, hepatic sections were dehydrated in ascending grades of ethanol and embedded in paraffin. Then, the sections were stained with hematoxylin and eosin (H & E) and observed for histopathological changes using the light electric microscope (Leika DMRBE, Biberach, Germany) using the DPController software.

### 4.11. Statistical Analysis

Using Statistical Package for the Social Sciences (SPSS version 16.0, IBM, Chicago, IL, USA), data were analyzed by one-way analysis of variance (ANOVA), followed by post hoc Tukey’s test, and expressed as mean ± standard deviation. *p* < 0.05 was considered statistically significant.

## 5. Conclusions

In conclusion, these results elucidate that the combination treatment with EUG and AT-MSCs, in rats, strongly inhibited the advancement of CCl_4_-induced hepatic fibrosis, compared with AT-MSCs alone. Therefore, the future use of this combination could exert an efficient and novel therapeutic strategy for patients with hepatic fibrosis. Future clinical studies and increasing the knowledge for the therapeutic mechanisms are required to increase our perception of the effects of EUG on the antifibrotic potential of AT-MSCs.

## Figures and Tables

**Table 1:** The immunoreactive score (IRS) calculation (IRS: A × B = 0–12).

A (Percentage of Positive Cells)	B (Intensity of Staining)	IRS Score (A × B)
0 = no positive cells	0 = no color reaction	0–1 = negative
1 = <10% of positive cells	1 = mild reaction	2–3 = mild
2 = 10%–50% positive cells	2 = moderate reaction	4–8 = moderate
3 = 51%–80% positive cells	3 = intense reaction	9–12 = strongly positive
4 = >80% positive cells		

**Table 2:** Primers used in quantitative real-time polymerase chain reaction (PCR) assay.

Primer	Sequence of the Primer
***type 1 collagen***	Forward: 5′-CAT GTT CAG CTT TGT GGA CCT-3′
	Reverse: 5′-GCA GCT GAC TTC AGG GAT GT-3′.
***α-SMA***	Forward: 5′-CGA TAG AAC ACG GCA TCA TCA C-3′
	Reverse: 5′-GCA TAG CCC TCA TAG ATA GGC A-3′.
***TGF-β1***	Forward: 5′-GGA CTC TCC ACC TGC AAG AC-3′
	Reverse: 5′-GAC TGG CGA GCC TTA GTT TG-3′.
***GAPDH***	Forward: 5′-AGA CAG CCG CAT CTT CTT GT-3′
	Reverse: 5′-TGA TGG CAA CAA TGT CCA CT-3′.
